# Cortical and Striatal Circuits in Huntington’s Disease

**DOI:** 10.3389/fnins.2020.00082

**Published:** 2020-02-06

**Authors:** Sonja Blumenstock, Irina Dudanova

**Affiliations:** ^1^Department of Molecules – Signaling – Development, Max Planck Institute of Neurobiology, Martinsried, Germany; ^2^Molecular Neurodegeneration Group, Max Planck Institute of Neurobiology, Martinsried, Germany

**Keywords:** Huntington’s disease, cortex, basal ganglia, neural circuits, genetic mouse models, *in vivo* calcium imaging, optogenetics

## Abstract

Huntington’s disease (HD) is a hereditary neurodegenerative disorder that typically manifests in midlife with motor, cognitive, and/or psychiatric symptoms. The disease is caused by a CAG triplet expansion in exon 1 of the huntingtin gene and leads to a severe neurodegeneration in the striatum and cortex. Classical electrophysiological studies in genetic HD mouse models provided important insights into the disbalance of excitatory, inhibitory and neuromodulatory inputs, as well as progressive disconnection between the cortex and striatum. However, the involvement of local cortical and striatal microcircuits still remains largely unexplored. Here we review the progress in understanding HD-related impairments in the cortical and basal ganglia circuits, and outline new opportunities that have opened with the development of modern circuit analysis methods. In particular, *in vivo* imaging studies in mouse HD models have demonstrated early structural and functional disturbances within the cortical network, and optogenetic manipulations of striatal cell types have started uncovering the causal roles of certain neuronal populations in disease pathogenesis. In addition, the important contribution of astrocytes to HD-related circuit defects has recently been recognized. In parallel, unbiased systems biology studies are providing insights into the possible molecular underpinnings of these functional defects at the level of synaptic signaling and neurotransmitter metabolism. With these approaches, we can now reach a deeper understanding of circuit-based HD mechanisms, which will be crucial for the development of effective and targeted therapeutic strategies.

## Introduction

Huntington’s disease (HD) is a devastating movement disorder that affects about 1 in 10,000 people. Among the heterogeneous group of neurodegenerative diseases, it takes a special role based on its strictly genetic cause, i.e., an autosomal dominant mutation of the huntingtin (*HTT*) gene on chromosome 4 (The Huntington’s Disease collaborative research group, 1993). The repetition of a CAG codon above a number of 35 translates into an expanded polyglutamine (polyQ) tract in the HTT protein, and causes a cascade of pathological events manifesting in psychiatric, cognitive and motor symptoms. The disease usually starts in midlife, with age of onset inversely correlating to CAG repeat number (Ross et al., 2014), and follows the course of three consecutive stages. The initial stage is typically characterized by mood disorder, cognitive deficits, and subtle motor impairments. In the second stage, excessive, abrupt, and involuntary movements (chorea) become the dominant symptom, while motor skills such as gait, swallowing, and speech rapidly deteriorate. Cognitive capacities also continue to decline, culminating in dementia. In the third stage, severe weight loss and overall deterioration of health occurs and choreic movements are replaced by bradykinesia and rigidity. Finally, death becomes imminent 15 to 20 years after disease onset.

Pathologically, HD is characterized by neurodegeneration of the basal ganglia, which is particularly severe in the striatum. Prominent atrophy also occurs in the neocortex, the main input region of the striatum, and in advanced disease stages other brain regions become affected as well (Waldvogel et al., 2015). Striatal atrophy mainly results from the loss of GABAergic spiny projection neurons (SPNs), also known as medium spiny neurons, while cortical neurodegeneration is most pronounced in the motor and premotor areas and primarily affects cortical pyramidal neurons (CPNs), also referred to as principal cells. Importantly, changes in neuronal function occur long before overt cell death is observed, suggesting that circuit alterations underlie the early symptoms of the disease.

In this review we will outline the current understanding of circuit mechanisms of HD based on investigations in available genetic mouse models. As classical electrophysiological studies in HD models have been extensively reviewed elsewhere (Raymond et al., 2011; Galvan et al., 2012; Bunner and Rebec, 2016; Plotkin and Goldberg, 2018), our main emphasis will be on the most recent developments in the field enabled by technological advances in circuit analysis, such as long-term *in vivo* imaging, *in vivo* multi-channel electrophysiology, optogenetics, and systems approaches for unbiased characterization of transcriptomic and proteomic changes. With these tools at hand, in the next few years it should be possible to not only accurately describe the HD-related defects in cortical and basal ganglia circuits, but also attempt to ameliorate them through cell type-specific activity manipulations.

## Genetic Mouse Models of HD

A number of HD mouse models have been created over the years since the discovery of the causal mutation in the *HTT* gene. These models have been reviewed in detail elsewhere (Brooks and Dunnett, 2013; Pouladi et al., 2013), and here we will only highlight the ones that are most frequently used for the study of HD-related circuit defects (Table 1). They can be divided into truncated and full-length models, the latter including transgenic and knock-in lines. Truncated models are all transgenic and express an N-terminal fragment of HTT with a pathological polyQ stretch. The R6 lines were the first HD mouse lines to be generated and are among the best studied. The R6/2 mouse line contains ∼150 CAG repeats and shows an aggressive phenotype with very early neurophysiological, histological, and behavioral alterations and a lifespan of only ∼3–5 months (Mangiarini et al., 1996).

**TABLE 1:** Selected genetic HD mouse models*.

**Model**	**Type of genetic manipulation**	**References**
R6/2	Transgenic, human exon 1 fragment with ∼150 CAG repeats under human HTT promoter	Mangiarini et al., 1996
YAC128	Transgenic, human full-length HTT with 128 CAG repeats	Slow et al., 2003
BACHD	Transgenic, human full-length HTT with 97 mixed CAA-CAG repeats	Gray et al., 2008
CAG140	Knock-in, chimeric mouse/human exon 1 with 140 CAG repeats inserted into the murine Htt locus	Menalled et al., 2003
zQ175	Knock-in derived from the CAG140 line, 188 CAG repeats	Menalled et al., 2012

*This table is not intended to give a complete overview of existing mouse models of HD, but only contains the models widely used for circuits studies.

Transgenic full-length models of HD express full-length human mutant HTT (mHTT) and generally exhibit a slower disease progression than truncated models. The yeast artificial chromosome (YAC) transgenic strategy was used to generate several mouse lines, named corresponding to their number of CAG repeats: YAC18 (control), YAC46, YAC72, and YAC128 (Hodgson et al., 1999; Slow et al., 2003). The YAC128 mouse exhibits striatal followed by cortical atrophy and mimics human disease progression by displaying first a hyperkinetic and later a hypokinetic phenotype (Slow et al., 2003). The bacterial artificial chromosome (BAC) HD model carries 97 mixed CAA-CAG repeats, shows reduced cortical and striatal volume and progressive motor impairments (Gray et al., 2008).

Knock-in HD mouse models provide stronger construct validity than transgenic models, as the CAG expansion is inserted into the native murine *Htt* locus, thereby more closely resembling the genetic context of HD patients. In these mice, brain atrophy and motor defects slowly emerge in a protracted manner. Among the knock-in models are the HD allelic series mice with various CAG tract lengths, including CAG140 and the widely used zQ175 line (Menalled et al., 2002, 2003, 2012; Heikkinen et al., 2012). Although no model perfectly reproduces all the aspects of the human disease, the major findings on circuit phenotypes have been quite consistent between various transgenic and knock-in lines, strengthening the confidence that mouse models can deliver important insights into pathogenic mechanisms of this disorder.

## Circuits Affected in HD

**FIGURE 1:**
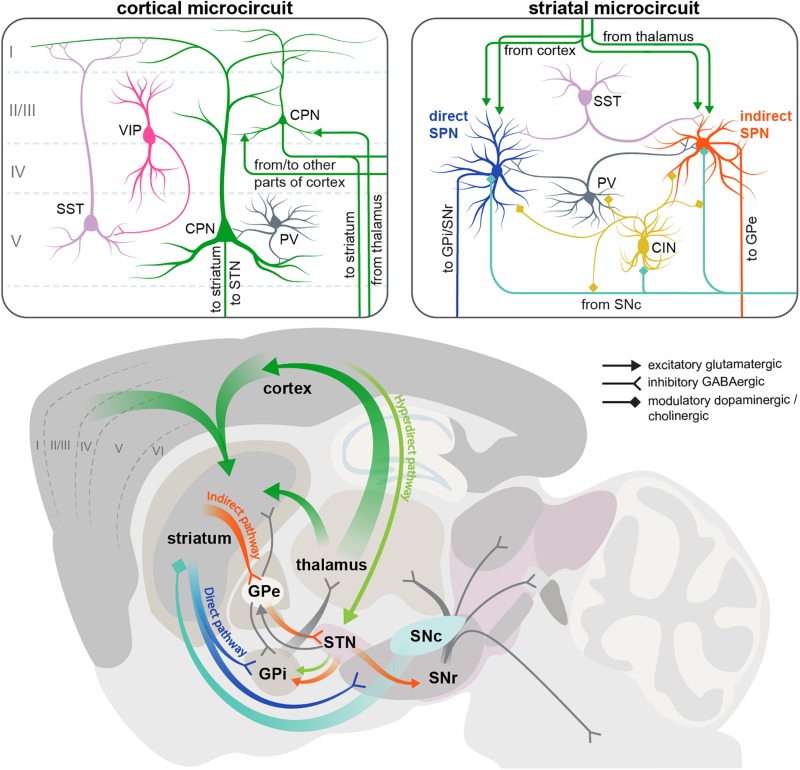
Scheme of the cortical and striatal circuits involved in HD pathogenesis. Two insets on the top show the main elements of the local cortical and striatal microcircuits. Roman numerals indicate cortical layers. For simplicity, only some of the cell types and connections between them are shown. CIN, cholinergic interneuron; CPN, cortical pyramidal neuron; GPe, external segment of the globus pallidus; GPi, internal segment of the globus pallidus; PV, parvalbumin interneuron; SNc, substantia nigra pars compacta; SNr, substantia nigra pars reticulata; SPN, spiny projection neuron; SST, somatostatin interneuron; STN, subthalamic nucleus; VIP, vasointestinal peptide interneuron.

Two brain regions most vulnerable to HD are the basal ganglia and the neocortex, which are extensively connected to each other (Figure 1). The neocortex contains two major neuron types: CPNs, which constitute ∼80% of all cortical neurons, and interneurons, which account for the remaining 20% (Defelipe et al., 2013; Huang, 2014). CPNs are excitatory glutamatergic neurons with long-range projections connecting cortical areas to each other or to subcortical structures. Interneurons are inhibitory GABAergic cells with mostly local connections. Based on the almost non-overlapping expression of molecular markers, cortical interneurons are subdivided into three main populations with distinct morphology, electrophysiological properties, layer distribution and function: parvalbumin (PV)-positive, somatostatin (SST)-positive and 5HT3a-receptor-positive cells (Tremblay et al., 2016). PV cells are known to synapse onto or close to the soma of CPNs and exert very fast and strong inhibition onto their target cells (Pfeffer et al., 2013; Hu et al., 2014), whereas SST cells form synapses on more distal dendrites (Wang et al., 2004). 5HT3aR cells are very heterogeneous, with a major subclass of this population expressing the marker vasointestinal peptide (VIP). VIP cells preferentially synapse onto SST interneurons (Pfeffer et al., 2013) (Figure 1).

The basal ganglia are a group of subcortical structures including the striatum, globus pallidus (GP), ventral pallidum, substantia nigra (SN), and subthalamic nucleus (STN). Within the basal ganglia, striatum is the region receiving most of the long-range input, including extensive glutamatergic innervation from CPNs (Figure 1). Although these afferents come from virtually all cortical areas, and are involved in many types of sensory, cognitive and motor functions, here we will focus on the motor circuitry relevant for HD, including motor cortical areas and the dorsolateral striatum. Another source of glutamatergic afferents to the striatum is the thalamus. Apart from these excitatory inputs, striatum also receives abundant modulatory dopaminergic afferents from the substantia nigra pars compacta (SNc).

GABAergic SPNs account for > 90% of striatal neurons and are subdivided into two populations of approximately equal size, giving rise to the two main striatal projections. The D1 dopamine receptor-expressing SPNs form the direct pathway, and are therefore referred to as dSPNs. This pathway projects directly to the output nuclei of the basal ganglia: the internal part of the globus pallidus (GPi) and the substantia nigra pars reticulata (SNr). The D2 dopamine receptor-positive SPNs form the indirect pathway and are referred to as iSPNs. This polysynaptic pathway connects to the output nuclei indirectly via the external segment of the globus pallidus (GPe) and STN (Figure 1) (Alexander and Crutcher, 1990). GPi and SNr consist of GABAergic neurons that have pacemaker properties and maintain tonic activity, continuously inhibiting their target cells in the ventral anterior and ventral lateral nuclei of the thalamus (Gerfen and Surmeier, 2011; Plotkin and Goldberg, 2018). Thalamic nuclei in turn send glutamatergic projections to the frontal cortex, forming the cortico-basal ganglia-thalamo-cortical loop. The direct and indirect striatal projections have opposing effects on the activity of the GABAergic cells in the GPi and SNr, and thereby on the overall output of the basal ganglia. The direct pathway inhibits the GPi/SNr activity and therefore has a net excitatory effect on the thalamus and cortex, facilitating execution of motor programs. Conversely, the indirect pathway disinhibits the GABAergic neurons in the GPi/SNr, leading to reduced activity of the thalamic and cortical neurons, and suppression of undesired movements (Alexander and Crutcher, 1990; Gerfen and Surmeier, 2011). It should be noted that this simple model of two antagonistic striatal pathways has been refined in the recent years by *in vivo* studies demonstrating simultaneous activation of dSPN and iSPN cell clusters during motion initiation, as well as similar correlation of their activity with locomotor behavior, suggesting a more sophisticated functional arrangement of basal ganglia circuits than previously thought (Cui et al., 2013; Barbera et al., 2016; Klaus et al., 2017; Parker et al., 2018).

In addition to the direct and indirect pathways, a third, hyperdirect pathway exists that bypasses the striatum and connects the frontal cortex to the output nuclei via glutamatergic neurons of the subthalamic nucleus (STN) (Figure 1). Like the indirect projection, this pathway also has a net inhibitory action on the thalamus and cortex. However, it conveys signals faster than the indirect pathway, and is believed to be important for precise timing of motor program initiation (Nambu et al., 2002).

Apart from SPNs, striatal microcircuits include multiple groups of local interneurons: cholinergic interneurons (CINs) and several types of GABAergic cells that can be distinguished by the expression of molecular markers such as parvalbimun (PV), neuropeptide Y (NPY), neuropeptide Y/somatostatin/nitric oxide synthase (NPY/SST/NOS), calretinin (CR), and tyrosine hydroxylase (TH). Overall, striatal interneurons receive similar types of afferents as striatal SPNs (glutamatergic from cortex and thalamus, and dopaminergic from SNc), and provide feedforward inhibition onto SPNs, modulating their activity on different time scales; however, each interneuron subtype has distinct connectivity and physiology. Striatal PV interneurons are fast-spiking cells (hence also referred to as FS interneurons) that preferentially target the soma and proximal dendrites of SPNs, providing fast and strong inhibitory inputs. All other GABAergic interneurons fire at lower rates and form synapses on distal SPN dendrites (Straub et al., 2016; Plotkin and Goldberg, 2018). CINs in turn modulate the activity of GABAergic cells as well as SPNs (Figure 1). There are also multiple connections between different striatal interneuron subtypes that are just beginning to be uncovered (Lee et al., 2017; Plotkin and Goldberg, 2018).

**FIGURE 2:**
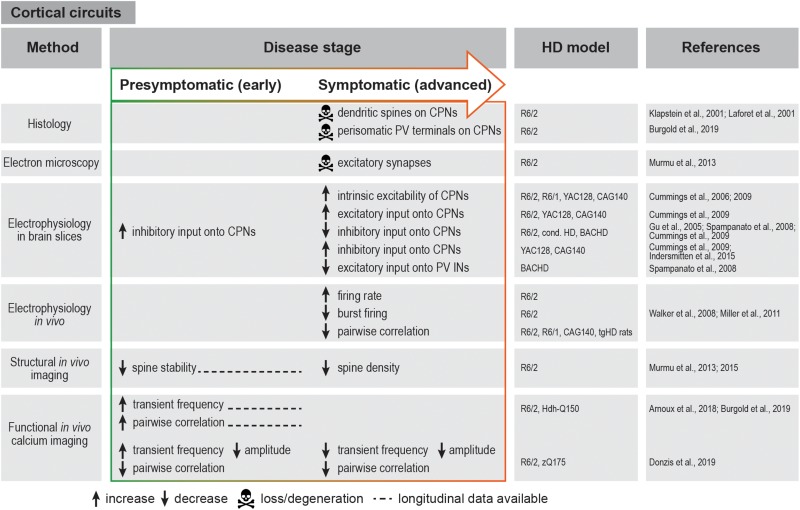
Summary of cortical circuit alterations in rodent HD models. cond. HD, conditional HD mouse model; CPN, cortical pyramidal neuron; IN, interneuron; PV, parvalbumin.

**FIGURE 3:**
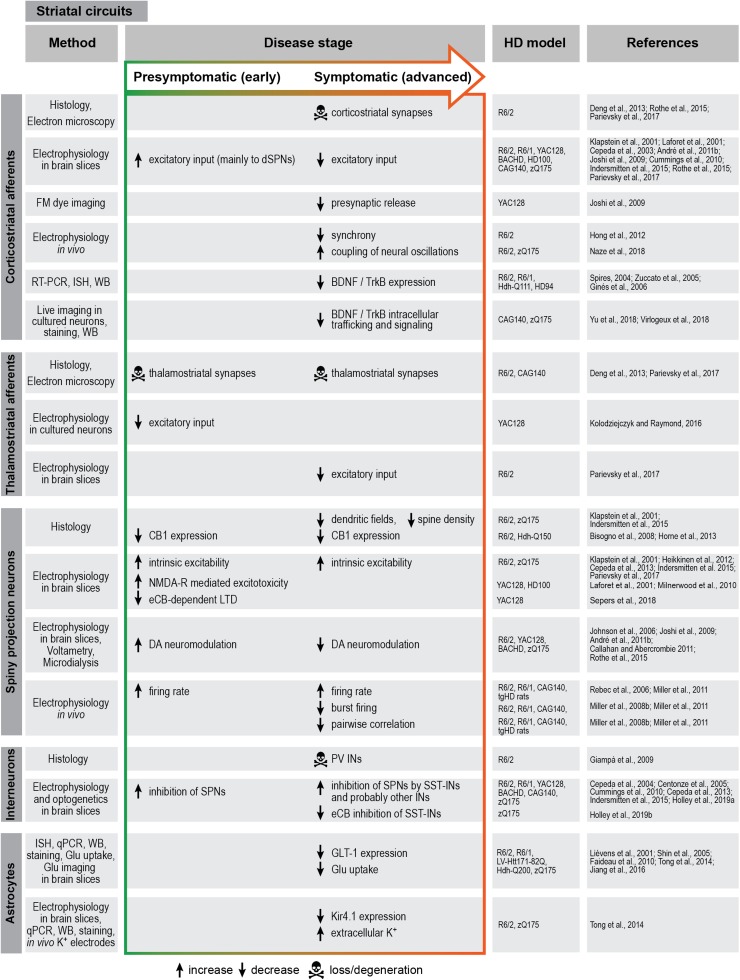
Summary of striatal circuit alterations in rodent HD models. BDNF, brain-derived neurotrophic factor; CB1, cannabinoid receptor 1; DA, dopamine; eCB, endocannabinoid; Glu, glutamate; HPLC, high-pressure liquid chromatography; IN, interneuron; ISH, *in situ* hybridization; LTD, long-term depression; NMDA-R, *N*-methyl-D-aspartate receptor; PV, parvalbumin; qPCR, quantitative polymerase chain reaction; RT-PCR, reverse transcription polymerase chain reaction; SPN, spiny projection neuron; SST, somatostatin; TrkB, tropomyosin receptor kinase B; WB, Western blot.

Although striatal interneurons provide the major source of GABAergic inhibition to SPNs, other inhibitory inputs also exist. Both dSPNs and iSPNs send collateral projections to other SPNs belonging to both pathways (Gittis and Kreitzer, 2012). Another layer of inhibitory connectivity is added by the reciprocal feedback projections between different nuclei of the basal ganglia that complement the unidirectional cortico-basal ganglia-thalamo-cortical loop described above (Plotkin and Goldberg, 2018). The following sections explain the specific defects that have been described in all these circuits during disease progression in various HD mouse models. A brief summary of these defects is given in Figures 2, 3.

### Cortical Circuits

Cortical degeneration and dysfunction significantly contribute to impairments in motor and executive functions and cognitive abilities observed in HD. This is underlined by detailed neuropathological studies in human post-mortem brains which revealed a reduction in overall cortical area and cortical white matter, associated with a marked cell loss. It is well-established that CPNs are particularly vulnerable to HD, however region-specific degeneration of interneurons also occurs (Kim et al., 2014; Mehrabi et al., 2016). Interestingly, neuronal cell loss in the cortex correlates with CAG repeat numbers (Halliday et al., 1998), as well as with the clinical symptomatology, such that patients with primarily motor symptoms show a prominent reduction in cell numbers in the primary motor cortex (Thu et al., 2010).

A growing body of evidence also highlights the fact that structural and functional alterations in the cortex precede neuronal loss by several years. The cortex of HD mutation carriers shows progressive regional thinning in a topographically predictable manner already up to 15 years before the onset of motor symptoms (Rosas et al., 2005, 2008; Nopoulos et al., 2010). On a functional level, one of the earliest events in HD is increased cortical excitability and impaired GABA-mediated cortical inhibition, as shown by transcranial magnetic stimulation studies already in the presymptomatic phase of the disease (Nardone et al., 2007; Schippling et al., 2009; Philpott et al., 2016; Agarwal et al., 2019).

Similar findings were also reported and further extended in HD mouse models, which exhibit multiple morphological and electrophysiological abnormalities of cortical neurons. Morphologically, dysmorphic dendrites and loss of dendritic spines on CPNs was observed in R6/2 and knock-in mice (Klapstein et al., 2001; Laforet et al., 2001). Chronic *in vivo* structural imaging furthermore demonstrated impaired spine turnover and a progressive loss of persistent spines in the somatosensory cortex (Murmu et al., 2013). Sensory deprivation exacerbated the loss of persistent spines and impaired stabilization of newly gained spines, suggesting that mHTT promotes maladaptive synaptic plasticity (Murmu et al., 2015).

Electrophysiological studies in HD mouse models revealed several changes in the basic membrane properties of CPNs, such as an increase in input resistance, decrease in cell membrane capacitance and depolarized resting membrane potential (Cummings et al., 2006, 2009; Stern, 2011). These alterations are known to affect synaptic plasticity and to make cells more excitable and therefore prone to excitotoxic damage. In addition to the elevated intrinsic excitability, there is an increase in excitatory inputs to CPNs. Thus, behaviorally phenotypic R6/2 mice present higher frequency of spontaneous excitatory post-synaptic potentials (EPSCs) and larger amplitudes of evoked EPSCs in layer II/III CPNs. Likewise, increased excitatory drive was also observed in the YAC128 and CAG140 models. Changes in inhibitory post-synaptic currents (IPSCs) seem to be more complex and variable between models: in R6/2 mice, IPSC frequency is initially increased in presymptomatic animals, but markedly declines at an advanced stage, while it is increased at an advanced stage in two different full-length mouse models (Cummings et al., 2009) (Figure 2).

*In vivo* electrophysiological recordings in R6/2 mice also showed faster and less variable firing rates in the medial prefrontal cortex. In addition, the firing pattern was temporally altered, with mostly individual spikes instead of coordinated bursts that typically occur in the WT cortex (Walker et al., 2008; Miller et al., 2011) (Figure 2). Although it should be noted that these parameters were not affected in knock-in HD mice, the observations in R6/2 animals are consistent with the possibility that the cortical activity is overall increased, but becomes “noisy” and less structured, possibly leading to impaired information processing in the cortex.

Recent advances in imaging techniques have allowed a more detailed investigation of cortical network dysfunction in large cell populations at single-cell resolution in a living animal. Using *in vivo* calcium imaging in transgenic and knock-in HD mice, we and others have demonstrated increased frequency of calcium transients at the premanifest disease stage and at disease onset, indicative of higher firing rates in CPNs as also suggested by electrophysiological recordings (Arnoux et al., 2018; Burgold et al., 2019; Donzis et al., 2019). In contrast, a decrease in calcium transient frequency and amplitude was observed in advanced-stage R6/2 and zQ175 knock-in mice (Donzis et al., 2019) (Figure 2). Although further studies spanning presymptomatic and symptomatic stages in the same mouse will be required to reconcile these observations, taken together, the presently available findings suggest dynamic changes in the cortical network during disease progression. An important advantage of chronic imaging is the possibility to longitudinally follow activity dynamics of single identified cells during disease course. This enabled us to show that a large fraction of neurons in the primary motor cortex of R6/2 mice become more active before the onset of motor symptoms, and maintain higher activity as disease progresses (Burgold et al., 2019).

In addition to the well-established perturbations of CPN activity, there is significant evidence supporting the involvement of cortical GABAergic interneurons in HD progression. Studies in conditional HD mouse models pointed to the importance of mHTT expression in cortical interneurons in addition to CPNs and the role of cell-cell interactions between CPNs and interneurons in the development of full-fledged cortical HD pathology and behavioral defects (Gu et al., 2005). Moreover, certain behavioral phenotypes were attributed specifically to cortical interneuron dysfunction (Dougherty et al., 2014). Electrophysiological recordings in HD mouse brain slices demonstrated reduced inhibitory inputs onto CPNs, along with reduced excitatory inputs onto PV interneurons and an altered probability of GABA release (Gu et al., 2005; Spampanato et al., 2008; Cummings et al., 2009). In agreement with these findings, a reduction in perisomatic PV terminals around CPNs was observed in both R6/2 mice and *post-mortem* HD patient tissue (Burgold et al., 2019). Altogether, these studies suggest that weakened inhibition might play a role in HD-related cortical network dysfunction. It should be noted that apart from the few studies on PV interneurons (Spampanato et al., 2008; Dougherty et al., 2014), the contribution of other major cortical interneuron types to HD is still largely unexplored.

The relevance of understanding interneuron function in HD becomes even more apparent when we appreciate their role in fine-tuning the activity of neuronal networks. GABAergic neurons promote fast spike synchrony between excitatory neurons and serve as pacemakers for generating neuronal oscillations, temporally defined cortical rhythms produced by coordinated activity of the network and important for cognitive functions. Thus, autaptic self-connectivity among PV neurons drives cortical oscillations in the gamma frequency range (Connelly, 2014; Deleuze et al., 2019). SST interneuron in turn are important for beta oscillations, and drive long-distance coherence across cortical areas (Chen et al., 2017; Veit et al., 2017). Interneurons are also believed to sculpt the functional flexibility of cortical circuits, which is a key factor for shaping behavior (Berke et al., 2004; Buzsáki et al., 2012; Cardin, 2019).

Studies in HD mouse models reach to some degree contradictory conclusions concerning cortical network synchrony assessed by correlations of simultaneously recorded neuron pairs. *In vivo* electrophysiology demonstrated reductions in spike synchrony in both transgenic and knock-in HD models regardless of disease stage (Walker et al., 2008; Miller et al., 2011; Stern, 2011), and implicated these deficits in behavioral alterations (Walker et al., 2011). While reduced pairwise synchrony was also observed in one *in vivo* calcium imaging study (Donzis et al., 2019), other studies described an increase in pairwise neuronal correlation as one of the earliest alterations in both transgenic and knock-in HD mice (Arnoux et al., 2018; Burgold et al., 2019). These initial insights call for further investigation to clarify the spatial and temporal manner in which network synchrony is affected in HD.

### Corticostriatal Projection

Changes in the corticostriatal connections are among the earliest events in disease progression, which occur before any signs of cell death can be detected, and presumably underlie the subtle motor deficits in premanifest HD (Reading et al., 2004; Unschuld et al., 2012; Hintiryan et al., 2016; Reiner and Deng, 2018). Disconnection from cortical afferents likely plays a major role in the subsequent dysfunction of the downstream striatal circuits. The breakdown of corticostriatal communication in HD has been extensively studied, and for detailed information the reader is referred to several excellent reviews available on this topic (Miller and Bezprozvanny, 2010; Raymond et al., 2011; Estrada-Sánchez and Rebec, 2013; Plotkin and Surmeier, 2015; Bunner and Rebec, 2016; Rebec, 2018). The conclusion that emerged from electrophysiological studies in slices as well as *in vivo* in multiple HD mouse models is that alterations in corticostriatal connections occur in two phases, with increased glutamate release and SPN hyperexcitation at the presymptomatic stage, followed by SPN silencing at the symptomatic stage (Figure 3) (Klapstein et al., 2001; Cepeda et al., 2003; Rebec et al., 2006; Joshi et al., 2009; André et al., 2011b; Miller et al., 2011; Raymond et al., 2011; Indersmitten et al., 2015; Rothe et al., 2015). The elevated cortical activity observed early in disease causes an increased excitatory drive onto striatal SPNs. Excess glutamate release from the cortical synaptic terminals leads to sustained activation of extrasynaptic NMDA receptors, triggering apoptotic mechanisms in the SPNs (DiFiglia, 1990; Okamoto et al., 2009; Milnerwood et al., 2010). Conversely, the decrease in SPN activity at a later stage is due to progressive loss of cortical inputs. Morphological investigations show a decrease in corticostriatal synaptic terminals in symptomatic animals (Deng et al., 2013), as well as progressive decline in spine densities on SPN dendrites (Indersmitten et al., 2015).

*In vivo* electrophysiological studies in freely behaving mice also analyzed local field potentials (LFPs), transient extracellular signals generated by large populations of neurons. These recordings uncovered altered synchrony between the cortical and striatal networks in HD (Hong et al., 2012; Naze et al., 2018). However, it is still unknown how cortical activity shapes firing patterns in the striatum. A study combining wide-field calcium imaging in the cortex with simultaneous multielectrode recordings in the dorsal striatum elucidated some of the principles of corticostriatal activity coupling during a behavioral task (Peters et al., 2019). Such cellular resolution studies would be invaluable to obtain a more comprehensive picture of corticostriatal miscommunication in HD.

An important function of the corticostriatal afferents is providing trophic support for SPNs, which are dependent on brain-derived neurotrophic factor (BDNF) for survival. BDNF is mainly produced in the cortex and delivered to the striatum anterogradely via CPN axons (Altar et al., 1997). mHTT decreases the levels of BDNF and its receptor tropomyosin-related kinase B (TrkB) in human (Ferrer and Blanco, 2000; Zuccato, 2001) and mouse brain (Spires, 2004; Zuccato et al., 2005; Ginés et al., 2006). Moreover, impaired transport and reduced release of BDNF has been observed in cortical neurons of zQ175 mice (Yu et al., 2018). Genetic reduction of BDNF in mice leads to striatal degeneration and expression profile similar to human HD, arguing for a major contribution of insufficient trophic support to striatal degeneration in HD (Strand et al., 2007).

Abundant evidence of corticostriatal miscommunication and impaired BDNF trophic support in HD obtained from studies in brain slices and *in vivo* was also confirmed in a recent *in vitro* approach using compartmentalized microfluidic chambers to reconstitute corticostriatal connectivity in a dish (Virlogeux et al., 2018). Consistent with previous findings, this study highlighted the important contribution of cortical afferents to the functional alterations observed in the post-synaptic striatal neurons.

It should be noted that in addition to the corticostriatal projection, the glutamatergic thalamostriatal afferents are also affected in various HD mouse models (Figure 3) (Kolodziejczyk and Raymond, 2016; Parievsky et al., 2017) and might be impaired even earlier than the cortical inputs (Deng et al., 2013). However, our knowledge about their significance in disease is still scarce.

### Basal Ganglia Circuits

Among the striatal cells, iSPNs are the most vulnerable to mHTT and are the first ones to degenerate (Reiner et al., 1988; Deng et al., 2004). This results in disinhibition of the thalamic target neurons, which is believed to underlie the hyperkinetic symptoms in HD. At a later stage, when dSPNs also succumb to disease, dyskinesia is replaced by akinesia and muscle stiffness (Albin et al., 1989; Plotkin and Goldberg, 2018; Reiner and Deng, 2018). Long before overt cell death occurs, SPNs start showing functional abnormalities. Some of the well-established early alterations in SPNs across various HD models are a depolarized membrane potential, an increase in input resistance, and hyperexcitability (Klapstein et al., 2001; Cepeda et al., 2003, 2013; Raymond et al., 2011; Heikkinen et al., 2012; Indersmitten et al., 2015; Dvorzhak et al., 2016; Parievsky et al., 2017). In addition, the two types of SPNs exhibit differential changes in glutamatergic, GABAergic inputs, and dopaminergic modulation, leading to an overall increased activation of dSPNs in particular (André et al., 2011a, b; Galvan et al., 2012; Deng et al., 2014). These functional changes are believed to be crucial to the imbalance of the direct and indirect pathway early in disease. It should be noted that recent demonstration of overlapping dSPNs and iSPNs activity patterns *in vivo* during motion (Cui et al., 2013; Barbera et al., 2016; Klaus et al., 2017; Parker et al., 2018) raised the possibility that disturbances occurring in HD likely go beyond a mere increase or decrease in the firing rates of one or both SPN types.

*In vivo* recordings of spontaneous activity in the striatum of freely behaving HD mice and rats revealed population-level impairments in striatal activity with a reduction in pairwise correlations and coincident bursts (Miller et al., 2008b, 2010). To obtain a more precise picture of these disturbances, it will be necessary to monitor the activity of different neuronal populations at a cellular resolution. However, while striatal circuits in HD models have been extensively explored in electrophysiological studies, their subcortical location has presented a certain difficulty for imaging. The development of head-mounted miniature microscopes that give optical access to deep brain structures should accelerate progress in this area (Werner et al., 2019). For example, calcium imaging in the striatum of a pharmacological mouse model of Parkinson’s disease revealed an imbalance of dSPNs and iSPN activity rates along with more complex changes in the spatiotemporal activity patterns and motion encoding that were specific to the iSPN population (Parker et al., 2018). It will be exciting to see what kind of changes can be uncovered by similar studies in freely behaving HD mice.

While the main focus in HD research has been on interplay between the direct and indirect pathways, more recent studies have also revealed abnormalities in the STN function that might contribute to motor impairments. *In vivo* extracellular recordings combined with electrocorticography demonstrated increased excitability of STN neurons in presymptomatic YAC128 animals, and reduced cortico-subthalamic coherence in symptomatic YAC128 and R6/2 mice. In addition, there was an overall decline in spontaneous activity and altered firing pattern of STN neurons (Callahan and Abercrombie, 2015a, b). These findings suggest that early hyperexcitability and later disconnection from the cortex is a general feature of cortico-basal ganglia projections in HD, affecting corticostriatal as well as corticosubthalamic pathways. Moreover, an *ex vivo* study in brain slices from two further mouse models, zQ175 and BACHD, confirmed reduced activity of STN neurons and attributed this reduction to an increased activation of NMDA receptors, resulting oxidant stress, and activation of K_*ATP*_ channels (Atherton et al., 2016). In addition to these functional changes, age-dependent loss of STN neurons was shown in human patients as well as HD mice (Lange et al., 1976; Guo et al., 2012; Atherton et al., 2016).

Striatal inhibitory circuits also show multiple defects in HD animals. Electrophysiological recordings demonstrated a consistent increase in GABAergic transmission onto SPNs of different mouse models, which in some cases occurred already at the presymptomatic stage (Cepeda et al., 2004, 2010, 2013; Centonze et al., 2005; Cummings et al., 2010; Indersmitten et al., 2015; Hsu et al., 2018). This increase in striatal inhibition, together with the loss of excitatory inputs, contributes to silencing of SPNs as disease progresses. Interestingly, the alterations in GABAergic inputs were distinct for the two types of SPNs, with a stronger effect in iSPNs found in three different mouse models at a symptomatic stage (André et al., 2011b; Galvan et al., 2012; Cepeda et al., 2013). These differences likely exacerbate the imbalance between the direct and indirect pathways.

Although the increase in inhibition onto SPNs is well-established, the underlying cell types and circuit mechanisms still remain to be deciphered. In contrast to SPNs, most local interneurons are relatively spared in HD, with the exception of PV interneurons, which are reduced in numbers in human patients and R6/2 mice (Giampà et al., 2009; Reiner et al., 2013; Reiner and Deng, 2018). A detailed analysis of PV cells in the zQ175 mouse model furthermore uncovered changes in morphology, physiological properties, and connectivity (Holley et al., 2019a). A recent study combining activity manipulation of PV interneurons with simultaneous calcium imaging of PV cells and SPNs in freely moving mice pointed to the function of PV cells in facilitating execution of movement (Gritton et al., 2019); it is therefore tempting to speculate that dysfunction and loss of striatal PV interneurons may play a causal role in HD-related akinesia, a hypothesis still to be tested in HD model animals.

Even though unchanged in numbers, other types of interneurons are also likely to be functionally affected in HD (Reiner and Deng, 2018). Recent studies started tackling the contributions of different interneuron types using genetic targeting and circuit manipulation tools (Cepeda et al., 2013; Holley et al., 2015, 2019b; Tanimura et al., 2016). Thus, optogenetic silencing of SST-positive interneurons [also referred to as low-threshold spiking (LTS) interneurons] in brain slices suggested a major contribution of this cell type to the increased GABAergic inhibition in symptomatic zQ175 and R6/2 mice (Holley et al., 2019b). In addition, the feedback inhibitory connections between SPNs are also partially severed, and their pattern is altered in HD mice, with a substantial number of abnormal bidirectional connections between SPN pairs (Cepeda et al., 2013). How this rewiring of SPNs contributes to their dysfunction remains a subject for future research.

Taken together, accumulated evidence points to biphasic alterations in SPNs as a result of multiple impairments in local and extrastriatal inputs: after initial hyperexcitation at the early stage, with disease progression the excitation/inhibition balance tips toward increased inhibition (Figure 3) (Galvan et al., 2012; Indersmitten et al., 2015).

## Neuromodulation by Dopamine and Endocannabinoids

### Dopaminergic Modulation

Alterations in glutamate-driven flow of information from the cortex to the striatum play a key role in the onset and progression of HD. Yet, the corticostriatal system is tightly regulated by dopamine (DA), a monoamine neuromodulator, which, together with GABA signaling, provides crucial counterbalance and adds flexibility to glutamatergic excitation of SPNs. In addition to the disturbances of glutamate and GABA signaling described above, many HD symptoms are therefore associated with altered dopaminergic modulation.

The dopaminergic circuit anatomy as well as dopaminergic signaling alterations in HD are described in detail in a number of excellent articles (André et al., 2010; Gerfen and Surmeier, 2011; Tritsch and Sabatini, 2012; Chen et al., 2013; Gardoni and Bellone, 2015; Rangel-Barajas and Rebec, 2016; Koch and Raymond, 2019). Two major dopaminergic pathways that innervate cortical and striatal areas and both show alterations in HD are the nigrostriatal and mesocorticolimbic pathways. The nigrostriatal pathway projects from the SNc to the dorsal striatum and is implicated in cognitive function and flexibility as well as in the control of movement (Bäckman and Farde, 2001; Groenewegen, 2003; Sleezer and Hayden, 2016; Andres and Darbin, 2018). In contrast, the mesocorticolimbic pathway originates from dopaminergic neurons in the ventral tegmental area (VTA) and ascends to the ventral striatum (or nucleus accumbens) and large areas of the frontal cortex. The mesocorticolimbic pathway plays a prominent role in motivation and reward-driven behavior.

DA can modulate the function of both excitatory CPNs and inhibitory SPNs on several levels, such as the probability of neurotransmitter release, the post-synaptic receptor sensitivity to the neurotransmitter (e.g., NMDA and AMPA receptors), and post-synaptic integration and ensuing excitability (Tritsch and Sabatini, 2012).

Intracellular DA signaling is mediated by a family of G-protein coupled receptors (D1–D5 DA receptors), which are grouped into two classes referred to as D1-like and D2-like. Upon receptor activation, one of the main targets of the recruited heterotrimeric G proteins is protein kinase A (PKA), which is positively or negatively coupled to D1- and D2-like receptors, respectively. As a consequence, the two classes of DA receptors drive complimentary cellular effects. The two types of SPNs show differential expression of DA receptors, with D1 receptors strongly enriched in dSPNs and D2 receptors in iSPNs (Tritsch and Sabatini, 2012). D1- and D2-like receptors exert opposite actions on presynaptic glutamate release and post-synaptic glutamate receptor currents: D1-like receptor signaling enhances glutamate release, depolarizes dSPNs, and increases their activity, while D2-like receptor signaling inhibits glutamate release, hyperpolarizes iSPNs, and leads to a decrease in their activity (Gerfen and Surmeier, 2011; Tritsch and Sabatini, 2012). An optimal level of dopaminergic modulation and balanced DA transmission between D1- and D2-like receptors is required for efficient motor function and behavioral flexibility (Chen et al., 2013).

In *post-mortem* HD brains, ∼40% dopaminergic neuron loss can be observed in the SNc along with a significant decrease in dopaminergic terminals in the striatum and loss of DA transporter (DAT) (Oyanagi et al., 1989; Suzuki et al., 2001). Transcriptional dysregulation of DA receptors in the striatum has also been reported in HD patients and HD models, with both D1- and D2-like receptors being reduced (Richfield et al., 1991; Weeks et al., 1996; Bibb et al., 2000; Ariano et al., 2002; Petersén et al., 2002; Pouladi et al., 2012).

During early disease progression, SPN loss is largely limited to striosomes (Hedreen and Folstein, 1995). As these cells project to the SNc, the death of these inhibitory neurons is thought to initially hyperactivate the nigrostriatal pathway, contributing to chorea and other clinical HD symptoms. First evidence for altered DA transmission came from the observation that pharmacologically increasing DA signaling in HD patients worsens chorea, whereas reducing DA leads to akinesia (Bird, 1980; Spokes, 1980). A wealth of following studies showed that progression of HD is accompanied by biphasic changes in DA inputs that are intertwined with changes in glutamate neurotransmission (Figure 3). The early symptomatic stage characterized by chorea is reflected by excessive glutamate and DA release, leading to a selective activation of the direct pathway and disinhibition of the thalamus. At a later stage, when chorea is replaced by hypoactivity, a lack of sufficient glutamate and DA signaling leads to the silencing of the direct pathway and inhibition of the thalamus (Johnson et al., 2006; Joshi et al., 2009; André et al., 2010, 2011b; Callahan and Abercrombie, 2011; Galvan et al., 2012; Rothe et al., 2015; Covey et al., 2016; Koch and Raymond, 2019).

Additionally, D2-mediated DA signaling onto striatal CINs generally reduces acetylcholine release and thereby dampens the inhibition of dSPNs. In HD models, despite the survival of CINs in the striatum, a dysregulation of acetylcholine release has been reported (Reiner and Deng, 2018). By this mechanism, increased levels of DA would exacerbate the imbalance toward activation of the direct pathway, and promote the development of HD symptoms (Smith-Dijak et al., 2019).

In cortical neurons, which receive both dopaminergic and glutamatergic input, a disturbed signal-to-noise ratio and reduced range over which DA and glutamate can be modulated impairs both cognitive and motor functions (Kiyatkin and Rebec, 1999; Dallérac et al., 2011). An interesting theoretical framework suggested that increased neural noise would therefore lead to inflexibility of brain activity and as a consequence, behavioral adaptations to environmental challenges would be impaired (Hong and Rebec, 2012).

Importantly, HD is often accompanied by a range of psychiatric symptoms, such as mood disorders, aggression, compulsive behavior, psychotic episodes, apathy, and sexual disorders (Roos, 2010; Tabrizi et al., 2013; Martinez-Horta et al., 2016). A lack of interest in life activities and depression are the most common mood symptoms of HD, appear early and continue during HD progression, thereby becoming one of the most disabling symptoms. This group of psychiatric symptoms has been linked to dysfunctional activity of PFC (Epping and Paulsen, 2011; Grace, 2016), which receives prominent dopaminergic input via the mesocorticolimbic pathway and plays a key role in reward. Impaired D1 receptor-mediated DA transmission was suggested to be involved in depression-like behaviors in HD (Renoir et al., 2012), and loss of function of D2-SPNs in the ventrolateral striatum causes motivational deficits without affecting spontaneous behavior or reward preference (Tsutsui-Kimura et al., 2017). Presymptomatic zQ175 mice further exhibit suppressed motivation to work for reward and compromised dopaminergic encoding of reward delivery in the nucleus accumbens (Covey et al., 2016).

### Modulation by Endocannabinoids

Endocannabinoids (eCBs), such as arachidonoylethanolamide (AEA) and 2-arachidonylglycerol (2-AG), are small lipophilic neuromodulators that are released from the post-synapse to diffuse locally and act retrogradely on presynaptic CB1 receptors where they inhibit neurotransmitter release. In the context of HD, decreased CB1 expression can be detected early in disease in both humans and animal models (Figure 3) (Denovan-Wright and Robertson, 2000; Glass et al., 2000; Dowie et al., 2009; Van Laere et al., 2010; Horne et al., 2013). Furthermore, changes in the levels of endogenous endocannabinoids have been reported (Bisogno et al., 2008), and CB1 knockout worsens motor performance in HD mice (Blázquez et al., 2011; Mievis et al., 2011). mHTT-dependent loss of CB1 furthermore disinhibits GABA neurotransmission in SPNs, and is associated with progressive decline of motor and cognitive function in HD models (Blázquez et al., 2011; Chiarlone et al., 2014).

eCB signaling plays important roles in synaptic plasticity at corticostriatal synapses. It modulates DA signaling to control flexible goal-oriented and reward-driven behavior (Hilário et al., 2007; Cui et al., 2015; Gremel et al., 2016; Augustin and Lovinger, 2018), two processes that are compromised in HD (Lawrence et al., 1996; Curtin et al., 2015). In the striatum, eCBs drive long-term depression (LTD) at both excitatory and inhibitory synapses (Huang et al., 2001). Striatal DA signaling, in contrast, is not modulated directly by eCBs, but is disinhibited indirectly via decreased GABAergic release at CB1-expressing inhibitory afferents. A recent study showed that accumbal eCB signaling inhibits CIN-driven DA release, whereas CIN activation recruits production of 2-AG, thereby providing negative feedback. Critically, 2-AG mobilization modifies DA-dependent reward-driven behavior (Mateo et al., 2017), and it has been shown that motivational deficits in HD mouse models can be normalized by pharmacologic elevation of 2-AG signaling at CB1 receptors (Covey et al., 2018). Additionally, attenuated LTD at corticostriatal synapses could be restored by inhibiting the degradation of 2-AG (Sepers et al., 2018), and viral delivery of CB1 ameliorated some of the cellular dysfunction observed in R6/2 mice (Chiarlone et al., 2014; Naydenov et al., 2014).

Activation of CB1 receptors seems to be neuroprotective, possibly via inducing expression of BDNF (Blázquez et al., 2015). It is furthermore suggested to play a role in the differential vulnerability of iSPNs vs. dSPNs by protecting dSPNs in particular (Ruiz-Calvo et al., 2018), although the mechanism of this selectivity is not yet clear. Therefore, stabilization of dopaminergic and endocannabinoid neuromodulatory systems are attractive targets for novel drugs treating HD.

## Role of Astrocytes in HD-Related Circuit Dysfunction

In addition to the changes in various neuronal cell types described above, astroglia has recently emerged as an important contributor to neuronal dysfunction in HD (Khakh et al., 2017). Indeed, mice selectively expressing mHTT in astrocytes exhibit age-dependent motor deficits and shortened life span (Bradford et al., 2009; Meunier et al., 2016). Astrocytic alterations have been so far mostly studied in the striatum, where astrocytes seem to play a crucial role in HD-related excitotoxicity via at least two interrelated mechanisms.

First, astrocytes are equipped with glutamate transporters and are responsible for the rapid clearance of extracellular glutamate released at synapses (Ransom and Ransom, 2012). A well-established HD phenotype in humans and mice is the loss of the astrocytic glutamate transporter GLT1 (also called EAAT2) (Arzberger et al., 1997; Liévens et al., 2001; Behrens, 2002; Shin et al., 2005; Faideau et al., 2010; Cao et al., 2019), which is probably due to transcriptional inhibition in the presence of mHTT (Bradford et al., 2009). Reduced expression of GLT-1 results in impaired glutamate uptake and increased extracellular glutamate levels (Figure 3) (Liévens et al., 2001; Behrens, 2002; Shin et al., 2005; Faideau et al., 2010; Estrada-Sánchez and Rebec, 2012). Accordingly, increasing GLT-1 expression rescues glutamate uptake and improves behavioral phenotypes in HD mice (Miller et al., 2008a). It should be noted that this well-established view was recently challenged by an *in vivo* imaging study of glutamate dynamics, which showed normal glutamate uptake in the striatum of HD mice (Parsons et al., 2016), a controversy that remains to be resolved.

Second, astrocytes play an important role in extracellular K^+^ buffering (Ransom and Ransom, 2012). This function is also disturbed in HD, as expression of mHTT leads to a downregulation of the Kir4.1 inwardly rectifying potassium channel on the cell membrane of astrocytes, causing a shift in the distribution of K^+^ ions across the membrane (Figure 3). The resulting increase in K^+^ concentration in the extracellular space is likely to be at least one of the underlying causes of depolarized membrane potential and elevated excitability of SPNs (Tong et al., 2014). In addition, it also depolarizes the membrane potential of astrocytes and reduces the electrogenic uptake of glutamate through the GLT-1 transporter, further impairing extracellular glutamate clearance (Dvorzhak et al., 2016). A recent imaging study with genetically encoded calcium and glutamate sensors in brain slices of HD mice not only confirmed the prolonged presence of extracellular glutamate after cortical stimulation, but also demonstrated profound alterations of Ca^2+^ signaling in astrocytes (Jiang et al., 2016). Interestingly, those defects could be partially rescued by restoring Kir4.1 expression in astrocytes, probably via improved GLT-1 expression and/or function (Dvorzhak et al., 2016; Jiang et al., 2016).

## Insights From Systems Biology Studies

While electrophysiology and imaging have been useful to describe the nature and time course of neural circuit dysfunction in HD models, the molecular links between the HTT mutation and neuronal miscommunication for a long time remained elusive. Recently, powerful systems biology approaches have provided important insights into these matters through large-scale and unbiased screens at the transcriptomic and proteomic levels. Next generation RNA-sequencing studies performed in HD mouse models and human HD patient induced pluripotent stem cell (iPSC)-derived or directly converted neural cultures have all highlighted dysregulation of synaptic genes. In particular, there was a downregulation of transcripts involved in the post-synaptic scaffold, neurotransmitter signaling, Ca^2+^ signaling, long-term synaptic plasticity, as well as reduced transcription of neuronal activity-regulated genes (Langfelder et al., 2016; HD iPSC Consortium, 2017; Veldman and Yang, 2018; Victor et al., 2018). Importantly, these changes also hold true at the proteomic level (Langfelder et al., 2016; Hosp et al., 2017; Skotte et al., 2018). Quantitative mass spectrometry analysis in R6/2 mice demonstrated a progressive decline of both excitatory and inhibitory synaptic proteins (Burgold et al., 2019), in line with morphological and functional defects described for both types of synapses. Interestingly, synapse-related proteins are also abundantly present in the mHTT interactome (Shirasaki et al., 2012) and within insoluble mHTT inclusion bodies (Hosp et al., 2017), suggesting that at least some of the synaptic defects are directly caused by the mutant protein, rather than being a secondary consequence of deteriorating neuronal health.

Systems biology studies furthermore deepened our understanding of astrocytic dysfunction in HD mice, not only by validating the reduction in glutamate transporters and K^+^ channels, but also by unveiling mHTT-induced metabolic disturbances in astrocytes (Langfelder et al., 2016; Skotte et al., 2018; Diaz-Castro et al., 2019). Among the altered astrocytic proteins were those involved in the glutamate-glutamine-GABA neurotransmitter metabolic cycle. Isotope labeling experiments in brain slices confirmed impaired astrocytic synthesis and/or release of glutamine, which serves as a precursor for GABA production in neurons and could play a role in the weakened striatal inhibition (Skotte et al., 2018).

## Open Questions and Future Directions

The impairments that occur in the principal neuron types in the cortex (CPNs) and striatum (SPNs) have been thoroughly characterized in the last two decades. Recent investigations have also highlighted the contribution of astrocytes to circuit dysfunction in HD. Despite these significant advances in the knowledge about circuit mechanisms of HD, major questions remain that will be particularly critical to address. The microcircuit mechanisms involving cortical and striatal interneurons are still largely unexplored. Moreover, it is not clear how low-level changes in synaptic connectivity and neuronal activity relate to higher-level changes such as network synchronization and LFP oscillations across brain areas. It is further not known whether and how such changes give rise to behavioral symptoms. Progress in this area will be facilitated by new techniques like multi-channel electrophysiology and deep brain imaging that permit simultaneous recording of large neuronal ensembles over prolonged periods of time. Manipulation of neuronal activity *in vivo* using optogenetic or chemogenetic approaches will be crucial to decipher the causal role of distinct circuit elements in pathomechanisms of HD.

Moreover, viewing the described cell types as homogenous populations is clearly an oversimplification. Single-cell RNA-sequencing (scRNA-seq) studies are revealing an ever-greater diversity of cortical neurons (Zeisel et al., 2015; Tasic et al., 2016, 2018) and challenging the classical subdivision of MSNs into two discrete D1 and D2 classes (Gokce et al., 2016). How this complex picture of cellular diversity is altered in diseases including HD is completely unknown. Extension of the scRNA-seq approach to HD models and human HD tissue will therefore be helpful to shed more light on the involvement of different neuronal and non-neuronal cell types and to tackle the enigmatic issue of differential vulnerability to mHTT.

*In vitro* co-culture systems have been useful in delineating the contribution of various afferents to SPN dysfunction (Kolodziejczyk and Raymond, 2016; Virlogeux et al., 2018). The availability of HD neural cultures either derived from iPSCs or directly converted from patients’ fibroblasts (HD iPSC Consortium, 2017; Victor et al., 2018) now opens the possibility of extending such *in vitro* approaches to human neurons, thus providing a convenient and physiologically relevant platform for drug testing. In addition, brain organoids (Lancaster et al., 2013) and further modifications of this technique that promote establishment of functional neuronal connectivity (Giandomenico et al., 2019) offer another promising way to bring *in vitro* modeling of disease-related circuit impairments closer to the *in vivo* setting.

At the molecular level, unbiased systems approaches including transcriptomics and proteomics have delivered important insights into the molecular underpinnings of neuronal dysfunction in HD. However, extensive further work will be required to link these molecular alterations to discrete functional deficits.

In conclusion, the development of novel methodologies allowing to monitor and manipulate neuronal activity in large populations of neurons during natural behavior, and to detect gene expression changes at a single-cell level, will enable a more complete understanding of circuit mechanisms of HD, and might open the doors for designing more refined therapies for this devastating disorder.

## Author Contributions

Both authors wrote and edited the manuscript.

## Conflict of Interest

The authors declare that the research was conducted in the absence of any commercial or financial relationships that could be construed as a potential conflict of interest.
